# Supplementary material for: Zibibbo Grape Seeds’ Polyphenolic Profile: Effects on Bone Turnover and Metabolism
Source: Pharmaceuticals (Basel). 2024 Oct 23;17(11):1418. doi: 10.3390/ph17111418 (PMC11597100; doi:10.3390/ph17111418)
Supplement: Supplementary file 1 [file pharmaceuticals-17-01418-s001.zip › pharmaceuticals-3240191-supplementary.pdf]

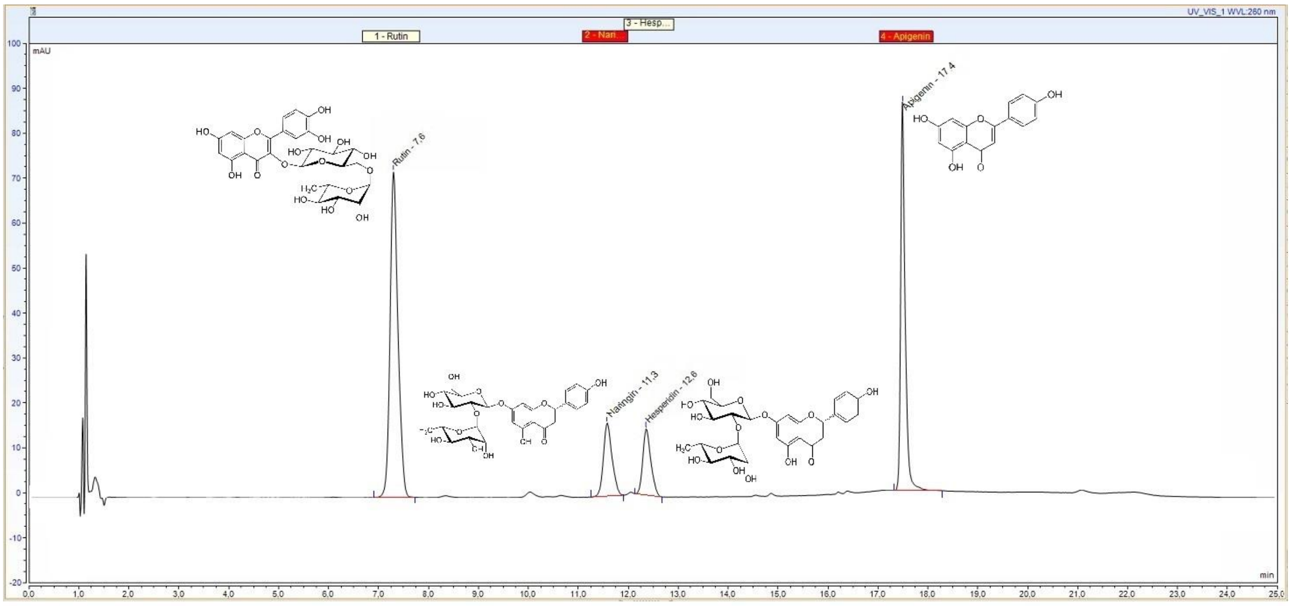


**Supplemental Figure S1 –** Chromatogram of standard flavonoids (rutin, naringin, hesperidin and apigenin) with their molecular structures.
